# High Hydrostatic Pressure Inducible Trimethylamine *N*-Oxide Reductase Improves the Pressure Tolerance of Piezosensitive Bacteria *Vibrio fluvialis*

**DOI:** 10.3389/fmicb.2017.02646

**Published:** 2018-01-09

**Authors:** Qun-Jian Yin, Wei-Jia Zhang, Xiao-Qing Qi, Sheng-Da Zhang, Ting Jiang, Xue-Gong Li, Ying Chen, Claire-Lise Santini, Hao Zhou, I-Ming Chou, Long-Fei Wu

**Affiliations:** ^1^Laboratory of Deep-sea Microbial Cell Biology, Institute of Deep-sea Science and Engineering, Chinese Academy of Sciences, Sanya, China; ^2^University of Chinese Academy of Sciences, Beijing, China; ^3^International Associated Laboratory of Evolution and Development of Magnetotactic Multicellular Organisms, CNRS-Marseille/CAS, Beijing, China; ^4^CAS Key Laboratory for Experimental Study under Deep-sea Extreme Conditions, Institute of Deep-sea Science and Engineering, Chinese Academy of Sciences, Sanya, China; ^5^Laboratory for Experimental Study under Deep-sea Extreme Conditions, Institute of Deep-sea Science and Engineering, Chinese Academy of Sciences, Sanya, China; ^6^LCB UMR 7283, CNRS-Marseille, Aix-Marseille Université, Marseille, France; ^7^Engineering Laboratory of Engineering Department, Institute of Deep-sea Science and Engineering, Chinese Academy of Sciences, Sanya, China

**Keywords:** high hydrostatic pressure (HHP), trimethylamine *N*-oxide (TMAO), pressure tolerance, *Vibrio fluvialis*, Raman spectrometry, CRISPRi

## Abstract

High hydrostatic pressure (HHP) exerts severe effects on cellular processes including impaired cell division, abolished motility and affected enzymatic activities. Transcriptomic and proteomic analyses showed that bacteria switch the expression of genes involved in multiple energy metabolism pathways to cope with HHP. We sought evidence of a changing bacterial metabolism by supplying appropriate substrates that might have beneficial effects on the bacterial lifestyle at elevated pressure. We isolated a piezosensitive marine bacterium *Vibrio fluvialis* strain QY27 from the South China Sea. When trimethylamine *N*-oxide (TMAO) was used as an electron acceptor for energy metabolism, QY27 exhibited a piezophilic-like phenotype with an optimal growth at 30 MPa. Raman spectrometry and biochemistry analyses revealed that both the efficiency of the TMAO metabolism and the activity of the TMAO reductase increased under high pressure conditions. Among the two genes coding for TMAO reductase catalytic subunits, the expression level and enzymatic activity of TorA was up-regulated by elevated pressure. Furthermore, a genetic interference assay with the CRISPR-dCas9 system demonstrated that TorA is essential for underpinning the improved pressure tolerance of QY27. We extended the study to *Vibrio fluvialis* type strain ATCC33809 and observed the same phenotype of TMAO-metabolism improved the pressure tolerance. These results provide compelling evidence for the determinant role of metabolism in the adaption of bacteria to the deep-sea ecosystems with HHP.

## Introduction

Trimethylamine *N*-oxide (TMAO) is widely dispersed in marine environments and plays an important role in the biogeochemical cycle of nitrogen (Ge et al., 2011). TMAO can be produced through oxidation of TMA by a variety of marine bacteria, phytoplankton, invertebrates and fishes (Barrett and Kwan, 1985; Seibel and Walsh, 2002; McCrindle et al., 2005). It accumulates in the tissue of marine animals and serves to protect against osmotic stress, adverse effects of low temperature, high concentration of urea and HHP (Yancey et al., 1982; Saad-Nehme et al., 2001; Zou et al., 2002; He et al., 2009; Petrov et al., 2012). The tissue concentration of TMAO increases proportionally with the depth where the fish lives, and the upper limit of the predicated isosmotic state created by TMAO at 8,200 m has been considered a biochemistry restriction that accounts for the absence of fish in the deepest 25% of the ocean (8,400–11,000 m) (Gillett et al., 1997; Yancey et al., 2014). TMAO can be metabolized by marine microorganisms through two pathways. It can be catabolized by the SAR11 clade and marine *Roseobacter* clade (MRC) bacteria as a carbon and nitrogen source (Lidbury et al., 2014, 2015) or as an electron acceptor of anaerobic respiration in diverse species of marine bacteria and most species of Enterobacteriaceae (Barrett and Kwan, 1985; Dos Santos et al., 1998; Dunn and Stabb, 2008).

The reduction of TMAO into TMA is mediated by the TMAO reductase system. The most extensively studied TMAO reductase system of *Escherichia coli* is composed of periplasmic TMAO reductase catalytic molybdo-subunits (encoded by *torA* or *torZ*), cytochrome C subunits (TorC or TorY) to transfer the electron from quinol to TorA, a chaperone TorD that is responsible for the folding and maturation of TorA and TorZ, a periplasmic protein TorT that is involved in TMAO sensing and a two-component regulatory system (TorSR) (Dos Santos et al., 1998; Genest et al., 2005). The expression of TMAO reductase is known to be induced only by the substrate. Binding of TMAO to TorT activates TorS, which subsequently phosphorylates TorR and starts the transcription of *torCAD* (Ansaldi et al., 2000, 2001; Baraquet et al., 2006).

Recent studies of deep-sea bacteria suggest that TMAO respiration might be involved in HHP adaptation. *Photobacterium profundum* piezophilic strain SS9 and piezophilic luminous bacterium *P. phosphoreum* ANT-2200 were isolated from 2,500 and 2,200 m depth, respectively (Nogi et al., 1998; Al Ali et al., 2010; Martini et al., 2013). They both encode multiple TMAO reductases in the genome, and expression of one of these genes in each strain is up-regulated under the HHP condition (Campanaro et al., 2005; Vezzi et al., 2005; Li et al., 2013; Zhang et al., 2016). Such HHP inducible TMAO reductase has never been reported in other species. It is speculated that the TMAO reductase might be constantly synthesized in the deep-sea piezosphere and thus facilitate quick reaction to TMAO released from fish and other deep-sea animals (Zhang et al., 2016). However, direct evidence supporting that TMAO respiration could be favorable for bacteria to live under HHP is lacking.

In this study, we isolated a piezosensitive strain of *Vibrio fluvialis* QY27 from 2,500 m depth of the South China Sea. We observed, for the first time, that supplementation of TMAO in the culture media can improve its cellular growth, especially under HHP, and conferred onto the bacterium a piezophilic-like phenotype. Two isozymes of TMAO reductase (TorA and TorZ) were identified in the genome of QY27. HHP enhanced the gene transcription and enzymatic activity of TorA only but not its homolog TorZ. Furthermore, by means of gene silencing, we confirmed that *torA* is responsible for the conspicuous TMAO-mediated piezophilic phenotype of this strain. A similar phenotype was also observed in the *Vibrio fluvialis* type strain ATCC33809. This is the first example showing the determinant role of energy metabolism in the HHP adaptation under deep-sea environments.

## Materials and Methods

### Isolation of Bacteria from Seawater Samples

Seawater samples were collected from 2,500 m depth from the South China Sea (E113°01.051′/N18°10.438′). A volume of 100 μL of seawater was inoculated into 2 mL YPG medium (Martini et al., 2013) and incubated at 30 MPa in high-pressure vessels overnight under ambient temperature before being plated on YPG agar medium for isolation of single colony. The 16S rRNA genes were amplified with 27F and 1492R primers, and the sequences were analyzed on the EzBioCloud database^1^ (Kim et al., 2012).

### Bacterial Strains and Culture Conditions

The *E. coli* strains were cultured at 37°C with a 160 rpm shaking speed. The diaminopimelic acid (DAP)-auxotroph *E. coli* strain WM3064 (for conjugation) was cultured with the supplementation of 0.3 M of DAP. *E. coli* strains that were transformed with plasmids pBBR1-MCS2/pCRISPRs, pdCas9_bacteria and pgRNA_RFP (purchased from Addgene) were maintained with 30 μg/mL kanamycin, 34 μg/mL chloramphenicol, and 100 μg/mL ampicillin, respectively. *Vibrio fluvialis* QY27 was cultured in YPG medium at room temperature (22–25°C). TMAO was supplemented to a final concentration of 1% (w/v) unless otherwise specified. The minimal medium used for the Raman spectrum analysis consisted of artificial seawater supplemented with vitamins, trace elements (Frankel et al., 1997) and HEPES (0.3%, w/v). Glycerol of 0.2% (v/v) was added to the minimal medium as the electron donor. QY27 strains carrying plasmids pCRISPRi+sgRNA*lux*/*torA/torZ* were cultured in YPG medium with 300 μg/mL kanamycin. Anhydrotetracycline (aTc) was added to a final concentration of 5 μM for the induction of Cas9. For the growth experiments, the cultivations were performed in 5 mL syringes filled with 3 mL media. The syringes were placed in high-pressure vessels (Feiyu Science and Technology Exploitation Co., Ltd., Nantong, China) and divided into two sets: one set was incubated at atmospheric pressure and another set at HHP. The hydrostatic pressure was applied with a water pump (Top Industrie, France). The cells were cultivated for 21 h in YPG medium, and samples were taken for the measurement of the absorption at 600 nm and protein contents every 3 h unless otherwise mentioned. The correlation between cell density and protein content was analyzed. The same sample used for the measurement of cell density was used for the total protein content assay. Cells from 2 mL culture volumes were re-suspended with 1 M NaOH and treated by boiling for 15 min. The protein content of the supernatant was quantified with Pierce BCA Protein Assay Kit (Thermo Fisher Scientific, Rockland, ME, United States). At least three independent experiments showed good correlation between the two sets of values under all conditions (Supplementary Figure S1), and the absorption at 600 nm was chosen as an indication of the biomass for the convenience in practice.

### Preparation of the Periplasmic Fraction

The periplasmic fractions were prepared by osmotic-shock treatment as previously reported (Ames et al., 1984). Briefly, QY27 cells of the mid-log phase were collected by centrifugation and re-suspend with Tris-HCl buffer (40 mM, pH 7.4) containing a proper amount of Complete Protease Inhibitor (Roche, Mannheim, Germany). Chloroform was added with gentle mixing and then incubated at room temperature for 5 min. A 10-times volume of Tris-HCl buffer was added and mixed by softly rotating the tubes 2–3 times. The samples were then immediately centrifuged and the aqueous phase was transferred into a clean tube and centrifuged one more time at 12,000 rpm and 4°C for 5 min. The protein quantification was performed with a Pierce BCA Protein Assay Kit (Thermo Fisher Scientific, Rockland, ME, United States).

### Analysis of TMAO Reductase Activity

TMAO reductase activity was measured by spectrophotometry. The anaerobic cuvette was filled with 4 mL of Tris-HCl previously bubbled with N_2_. The blank was measured after the addition of 10 μL of benzyl viologen. Then, Na dithionite dissolved in 0.2 M NaOH was added until the solution turned to dark blue. The absorption was recorded at 650 nm with a spectrophotometer after the addition of the periplasmic fraction. When the curve is flat, 20 μL of 2 M TMAO was added into the cuvette. The slope of decrease in absorption was recorded before and after the addition of TMAO. The Δslope is calculated by using the latter slope substrate and the former one. The specific TMAO reductase activity is defined as μmol BV oxidized per minute per mg of periplasmic protein and is calculated as a Δslope × 270.3/[protein concentration (mg/mL) × volume of periplasmic fraction (μL)].

### Detection of TMAO Reductase Isozymes

TMAO reductase isozymes were visualized by activity staining after resolving the periplasmic proteins on a native polyacrylamide gel, as previously reported (Santini et al., 1998). Unless otherwise specified, 6 μg of periplasmic protein was loaded on the gel for the cells cultured without TMAO and 0.06 μg for the cultures with addition of TMAO. The gel was first incubated in N_2_ bubbled Tris-HCl buffer containing methyl-viologen and Na dithionite until it was stained to dark blue. With the addition of TMAO, the protein band containing active TMAO reductase appeared white.

### RNA Extraction and Real-Time RT-PCR

Total RNA was extracted from the QY27 cells cultured to the mid-log phase. Briefly, approximately 10^7^ cells were collected and treated with trizol and chloroform. Isopropanol and ethanol were then used to precipitate and wash the nucleic acid before it was finally dissolved in 50 μL RNase-free water. Residual DNA was digested with DNase I. Reverse transcription was performed with a PrimeScript^TM^ II 1^st^ strand cDNA synthesis kit (TAKARA, Shiga, Japan). RT-PCR was conducted with StepOne Software (ABI) in reaction mixtures with a total volume of 20 μL and containing 10 μL of FastStart Universal SYBR Green Master (Rox) (Roche, Mannheim, Germany), 0.5 μM of primer, and 2 μL of cDNA template. The relative expression of the target gene was normalized to the reference gene of *rpoC*. Three replications were set for each assay for the calculation of the mean value and the standard deviation.

### Construction of Gene Silencing Strains

To construct plasmid pBBR1-MCS2-dCas9, DNA fragments of dCas9 and the pARO190 backbone were amplified by PCR from plasmids pdCas9_bacteria and pBBR1-MCS2, respectively, with a 70 bp overhang for each fragment. Primers 1 and 2 were used to amplify dCas9, and primers 3 and 4 were used to amplify pBBR1-MCS2; Primer 1: 5′-CTATGACCATGATTACGCCAAGCGCGCAATTAACCCTCACTAAAGGGAACAAAAGCTGGGTACCGGGCCCACGTCTTAAGACCCACTTTCAC-3′; Primer 2: 5′-CCGCGGTGGCGGCCGCTCTAGAACTAGTGGATCCCCCGGGCTGCAGGAATTCGATATCAAGCTTATCGATCAACAGATAAAACGAAAGGCCC-3′; Primer 3: 5′- GGGCCCGGTACCCAGCTTTTG-3′; and Primer 4: 5′-ATCGATAAGCTTGATATCGAATTCC-3′. A direct cloning method with RecET linear-linear recombineering was then used for assembly of the two fragments (Fu et al., 2012). To construct plasmid pCRISPRi + sgRNA*luxA*/*torA/torZ* (dCas9 + sgRNA), sgRNAs carrying the 20 nt specific targeting sequence were synthesized and inserted into the pBBR1-MCS2-dCas9 at GeneWiz Inc. (Suzhou, Jiangsu, China). The gRNA sequences were as follows: *luxA*: 5′-TCTTTATGTGACTCACCTGG-3′, *torA*: 5′-TGATTGGTGTCAGCAAGCTT-3′, and *torZ*: 5′-GTGTGCCCTGAGGTTTAGAT-3′. Plasmids pCRISPRi + sgRNA*luxA*/*torA/torZ* were transformed into the *E. coli* strain WM3064 by electroporation and then transferred into strain QY27 through bi-parental conjugation as described previously with slight modification of the culture condition (Rong et al., 2008).

### TMAO Detection by a Raman Spectrometer

The Raman spectrum analyses were performed using the LabRAM HR Evolution laser confocal micro-Raman spectrometer. The supernatant of the cultures was obtained by centrifugation at 14,000 rpm for 10 min and loaded on slides for Raman spectrum scanning. The spectra were collected for 30 s in the dark after the sample was excited by a laser at 532.06 nm (frequency-doubled Nd:YAG) with a 50× Olympus objective (0.25 numerical aperture) and a 1800 grooves/mm grating. The laser light with a power of approximately 20 mW was focused on the sample and the spectra were collected in the range from 50 cm^-1^ to 4000 cm^-1^. The results were analyzed by NGSLabSpec software.

For the real-time Raman spectrum analysis, the cells of QY27 were cultured in minimal medium with supplementation of 1% TMAO under 0.1 MPa and 30 MPa in the High-Pressure-Observation-cell (HPO-cell), which was designed and constructed by Syn Corporation, LTD (Kyoto, JAPAN) (Supplementary Figure S2A). An overnight culture of QY27 was inoculated into the fresh medium with inoculums of 1/100. Approximately 500 μL of the new culture was transferred into the HPO-cell and the lid was carefully sealed. Pressure was applied with a high-pressure pump from TOP Industrie if necessary. The HPO-cell was fixed on the microscope stage and incubated at room temperature for 47 h, and the Raman spectra were collected at the time indicated through the sapphire window (Supplementary Figure S2B).

To establish the TMAO and TMA quantification references, Raman spectra of the minimal medium supplemented with different concentrations of TMAO or TMA (0, 0.2, 0.4, 0.6, 0.8, 1.0, and 1.2%) were collected in the HPO-cell. The peak area was analyzed with GRAMS AI software. The peak area ratio is defined as the area value of the TMAO- or TMA-related peaks versus the internal reference peak r4, and the equations between the TMAO or TMA concentration and peak area ratio were set up.

## Results

### Growth Property of *Vibrio fluvialis* Strain QY27

**FIGURE 1:**
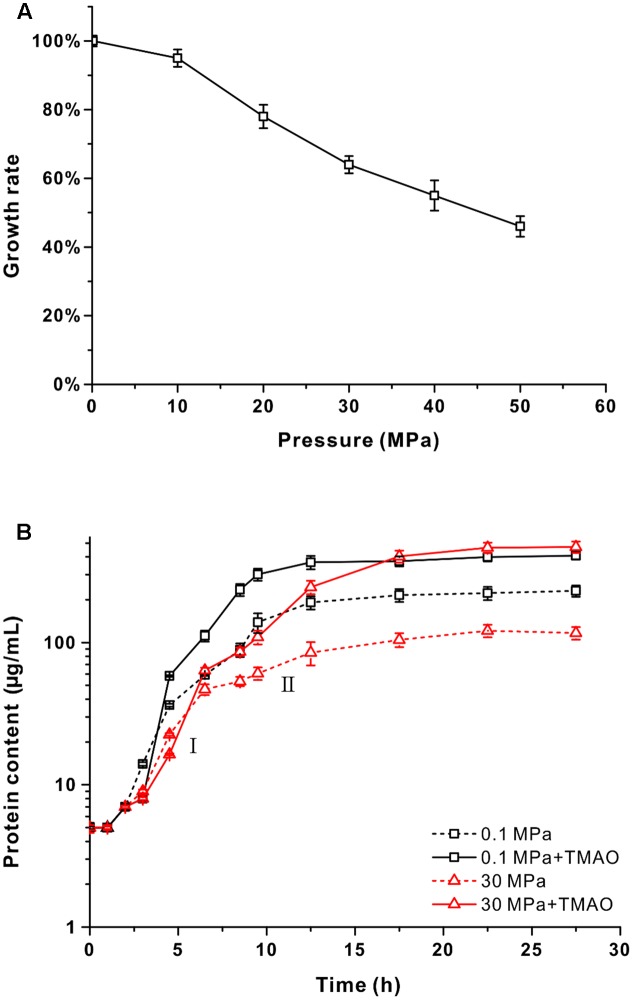
The effect of pressure and supplementation of TMAO on the growth of QY27. **(A)** The maximal growth rate of strain QY27 cultivated at different pressure conditions. The maximal growth rate at 0.1 MPa was set as 100%. **(B)** The growth curve of QY27 cultivated at different conditions. The dash lines represent cultures without TMAO, the solid lines represent culture with supplementation of TMAO. Lines in black represent cultures at 0.1 MPa, and lines in red represent cultures at 30 MPa. The protein content per milliliter culture is utilized to reflect the biomass. I and II indicate the first and the second growth phases of QY27 when cultivated at 30 MPa with supplementation of TMAO.

The QY27 strain was isolated from the seawater sample from 2,500 m depth from the South China Sea. Phylogenetic analysis based on the 16S rRNA gene sequence revealed that it shared 99.6% identity with the *V. fluvialis* type strain ATCC33809 isolated from human feces (Lee et al., 1981). Therefore, the QY27 strain is a different isolate of *V. fluvialis*. To evaluate whether QY27 was tolerant to HHP, we cultivated the cells at different pressure conditions from 0.1 to 50 MPa and measured the growth rates. As shown in **Figure 1A**, the optimal pressure for its growth was 0.1 MPa. When cultivated at 50 MPa, QY27 still grew but the growth rate was reduced to half compared to that at 0.1 MPa. Therefore, despite its origination from 2,500 m depth, QY27 is a typical piezo-sensitive strain.

### TMAO Improves the Pressure Tolerance of QY27

Several species of *Vibrio* were known to be able to utilize TMAO as an electron acceptor for anaerobic respiration (Proctor and Gunsalus, 2000). To test if QY27 was also capable of TMAO metabolism, the cells were cultivated in YPG medium at different pressures (0.1 and 30 MPa) with or without the supplementation of TMAO and the protein content was measured to reflect the biomass. At both 0.1 and 30 MPa, the addition of TMAO led to an increase of biomass but with different rates. At the atmospheric pressure, the biomass almost doubled with the presence of TMAO (0.37 ± 0.02 mg/mL versus 0.19 ± 0.02 mg/mL), whereas it increased by approximately sixfold at 30 MPa (0.46 ± 0.01 mg/mL versus 0.08 ± 0.01 mg/mL) and the final biomass even exceeded that at 0.1 MPa (**Figure 1B**, red line versus black line). A closer examination of the growth curves reveals that when cultured at 30 MPa, the addition of TMAO did not affect the maximal growth rate significantly but led to a diauxic growth. The growth rates at the first phase (around 2 – 7.5 h) were similar to other conditions, but a significant growth at the second phase (from 10 to 18 h after inoculation) was observed only for the cultures at HHP with TMAO (**Figure 1B**, red line, I and II). Similar results were obtained with the minimal medium in which glycerol and TMAO were used as a sole electron donor and acceptor, respectively (Supplementary Figure S3). Taken together, these results demonstrated that TMAO could improve the pressure tolerance of strain QY27 and change the piezo-sensitive phenotype to a piezophilic-like one. It should be noted that the same phenotype was observed in the *V. fluvialis* type strain ATCC33809 (Supplementary Figure S4), which is isolated from human feces. These results suggest that the beneficial effect of TMAO on the growth at the HHP condition is not specific only for strain QY27.

**FIGURE 2:**
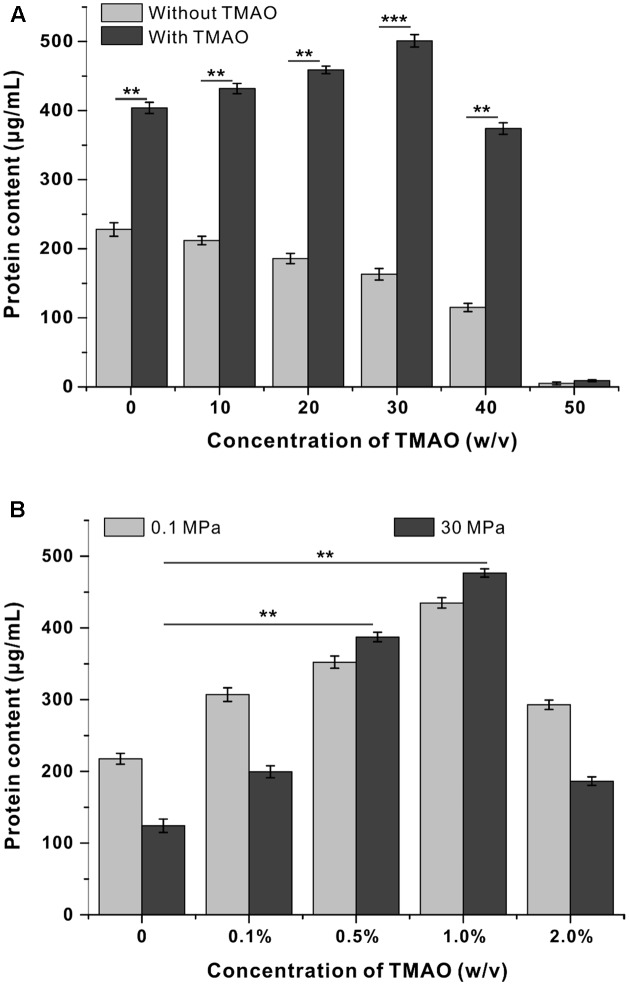
Influence of TMAO on the growth of QY27 at elevated pressure. **(A)** The effect of TMAO (1.0%, w/v) on the growth of QY27 at different pressure conditions. **(B)** The effect of TMAO of different concentration on the growth at 0.1 MPa and 30 MPa. The biomasses of different cultures were measured in stationary phase. The Student’s *t*-test was performed, ^∗^*P* < 0.05, ^∗∗^*P* < 0.01, ^∗∗∗^*P* < 0.001, and ns represents no significant difference.

To determine to what extent the pressure tolerance could be influenced by TMAO, QY27 was cultivated at even broader pressure conditions, and the biomass was measured at stationary phase. The growth of QY27 was clearly hampered by HHP as the biomass decreased along with the increase of pressure (**Figure 2A**, gray bars). However, with the addition of TMAO, the biomass increased progressively when the pressure increased from 0.1 to 30 MPa and then started to decrease at 40 MPa (**Figure 2A**, black bars). Thus, subsequent growth experiments at HHP conditions were performed at 30 MPa.

We further determined the optimal TMAO concentration for the growth of QY27. As shown in **Figure 2B**, under both 0.1 and 30 MPa conditions, TMAO with a concentration up to 1.0% (w/v) was beneficial for the growth of QY27. A further increase of the concentration of TMAO to 2.0% led to a decrease of the final biomass, which indicated a negative effect. It is noteworthy that the piezophilic-like phenotype was observed only when 0.5 or 1.0% TMAO was supplemented, whereas the cells remained piezosensitive with a lower (0.1%) or higher (2.0%) concentration of TMAO in the medium. Together, the results converge at the conclusion that a proper amount of TMAO improves the pressure tolerance of the piezosensitive strain QY27.

### TMAO-Improved Growth under High Pressure Relies on TMAO Respiration

**FIGURE 3:**
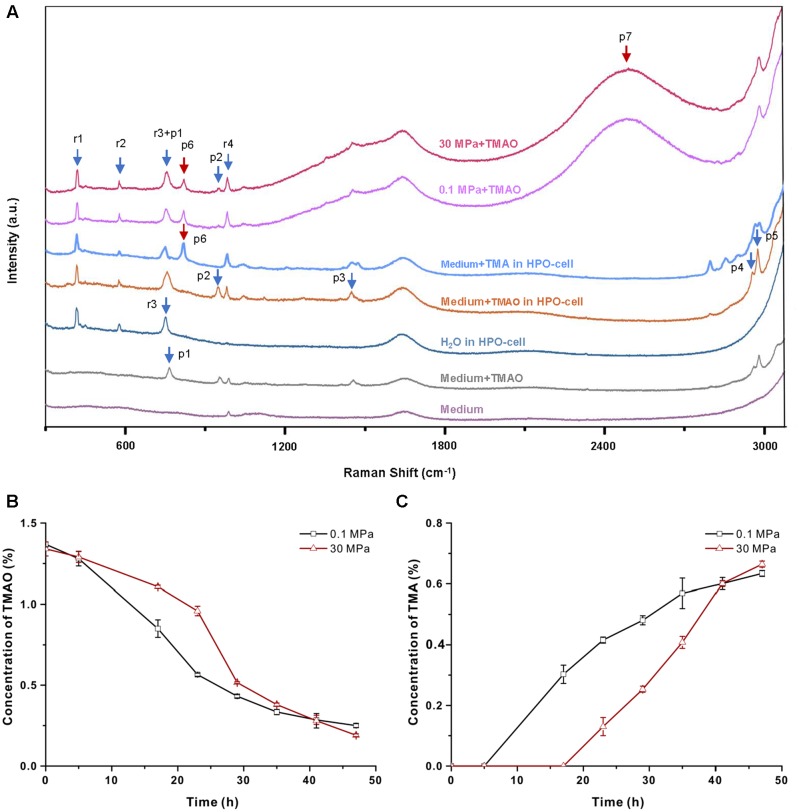
Raman spectrometry analysis of the utilization of TMAO. **(A)** Raman spectra of different solutions. From the bottom to top: minimal medium on slide, minimal medium with 1% TMAO on slide, water in HPO-cell, minimal medium with TMAO in HPO-cell, supernatant of QY27 cultured at 0.1 MPa and supernatant of QY27 cultured at 30 MPa. Arrows mark the TMAO-specific peaks (p1–p5), product of TMAO metabolism (p6 and p7) and unrelated peaks (r1–r4). **(B)** The concentration of TMAO in the supernatant of QY27 cultures at 0.1 MPa and 30 MPa. The concentration of TMAO was calculated by the ratio of peak area of p2/r4. **(C)** The concentration of TMA in the supernatant of QY27 cultures at 0.1 and 30 MPa. The concentration of TMA was calculated by the ratio of peak area of p6/r4.

To understand the mechanism of TMAO-improved pressure tolerance, we cultivated QY27 in a HPO-cell and monitored the TMAO metabolism in real time by means of Raman spectrometry (see section “Materials and Methods”). To reduce the background signal from the organic components in the culture medium, all of the experiments were performed using the minimal medium in which QY27 exhibited identical TMAO-improved growth at HHP (Supplementary Figure S3). As shown in **Figure 3A**, eight peaks can be observed when the minimal medium plus TMAO was analyzed in the HPO-cell. Among them, four peaks at 959 (p2), 1456 (p3), 2960 (p4) and 2988 (p5) cm^-1^ were originated from TMAO, three peaks at 417 (r1), 580 (r2) and 985 (r4) cm^-1^ were originated from the HPO-cell, and the peak called r3+p1 at 756 cm^-1^ was an overlap of peak r3 at 748 cm^-1^ and peak p1 at 765 cm^-1^. After the cultivation of QY27, the TMAO-related peaks (p2, p3, p4, and p5) decreased while two more peaks at 826 cm^-1^ (p6) and 2500 cm^-1^ (p7) progressively appeared and increased. Addition of TMA in the minimal media in HPO-cell led to several peaks including the peak p6 that has been identified as a typical band in the Raman spectra of gaseous TMA (**Figure 3A**) (Clippard and Taylor, 1969). Therefore, the peak p6 represented TMA, the direct product of bacterial TMAO reduction. The nature of p7 remained unclear.

To better understand the TMAO metabolism under atmospheric and HHP conditions, we first established the quantification of TMAO and TMA by measuring the areas of TMAO-related peak p2 and the TMA-related peak p6 at different concentrations (Supplementary Figures S5A,B). Using the peak r4 as an internal reference, we normalized the TMAO or TMA concentrations by the calculation of the area ratio of p2 (TMAO) or p6 (TMA) compared to the peak p4. We found that the ratio of the peak areas p2/r4 or p6/r4 were in proportion to the TMAO or TMA concentration in the media (Supplementary Figures S5C,D), which indicated a reliable correlation between the Raman signal and TMAO or TMA concentration and thus allowed monitoring of the reduction of TMAO under different pressure conditions.

Based on the time course the Raman spectra of the cultures at 0.1 and 30 MPa (Supplementary Figure S6), the concentrations of TMAO and TMA in the medium were calculated. As shown in **Figure 3B**, the concentration of TMAO started to decrease after 5-h of cultivation at 0.1 MPa. The consumption of TMAO in the cultures at 30 MPa was slightly postponed but at an accelerated rate (0.07% TMAO per hour during 23–29 h at 30 MPa versus 0.05% TMAO per hour during 17–23 h at 0.1 MPa). Fewer remnants of TMAO were observed after cultivation at 30 MPa compared to the cultures at 0.1 MPa. Accordingly, the appearance of TMA at 0.1 MPa was ahead of that at 30 MPa, but the rate of increase and the final concentration of TMA were higher at 30 MPa (**Figure 3C**). Together, the tendency of TMA production is consistent with that of TMAO consumption, and they both indicated a later but more efficient TMAO reduction at 30 MPa.

The Raman spectra analysis suggests that the reduction of TMAO into the TMA took place during growth. The bacterial reduction of TMAO can be catalyzed by both TMAO reductase and DMSO (Dimethyl sulfoxide) reductase. The former is highly specific for *N*-oxides including TMAO, whereas the latter recognizes both the *N*-oxides and *S*-oxides such as DMSO (Dos Santos et al., 1998). To clarify which enzyme took part in the TMAO metabolism of QY27, we analyzed the effect of DMSO on the growth. As shown in the Supplementary Figure S7 and unlike TMAO, the supplement of DMSO did not improve the growth, which indicated the absence of DMSO reductase in QY27. Therefore, the TMAO-improved growth at HHP seemed to be dependent on the TMAO reductase.

### Enhancement of QY27 TMAO Reductase Activity by Elevated Pressure

The physiological analysis and Raman spectrometry results suggest an implication of TMAO reductase in the TMAO-improved pressure tolerance of QY27 at high pressure. We corroborated this hypothesis by genetic and biochemistry analyses.

A complete TMAO reductase system was identified in the draft genome of QY27 (accession number PHIF01000000). It consisted of a *torECA* gene cluster, a *torYZ* cluster, a *torT-torS* cluster and a *torD-torR* cluster (Supplementary Figure S8). There were two genes encoding the catalytic subunit of TMAO reductase (*torA* and *torZ*). They shared 99% identity with the TorA and TorZ of the *V. fluvialis* type strain ATCC33809 and 37% and 70% identity to those of *E. coli*, respectively.

Similar to the homologues of *E. coli*, the TorA and TorZ proteins of QY27 possess the twin-arginine translocation (TAT) export signal peptides and therefore should be exported into the periplasm via the TAT pathway as previously reported for TorA in *E. coli* (Santini et al., 1998). TorA alone exhibits TMAO reductase activity *in vitro* when benzyl viologen or methyl viologen is used as an artificial electron donor for the reduction of TMAO. The periplasmic fraction containing TorA was prepared for the analysis of TMAO reductase activities by both the spectrophotometry-based enzyme assay and activity staining after resolving the proteins on native polyacrylamide gels. The enzyme assay allows the quantification of the total TMAO reductase activity, whereas the latter permits the visualization of different isozymes.

**FIGURE 4:**
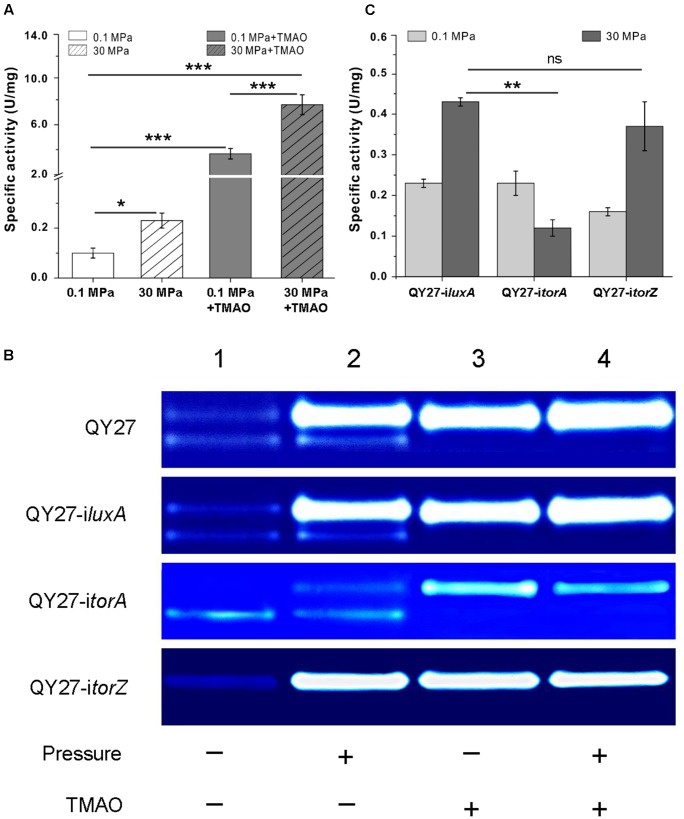
Effect of HHP on TMAO reductase activities. **(A)** The specific activity of TMAO reductase of QY27 cultivated at different conditions. **(B)** Isozyme staining of TMAO reductase of the wild type strain QY27 (QY27), QY27 carrying control plasmid (QY27-i*luxA*), *torA*-silencing strain (QY27-i*torA*) and *torZ*-silencing strain (QY27-i*torZ*). Lane 1, cultures at 0.1 MPa without TMAO; Lane 2, cultures at 30 MPa without TMAO; Lane 3, cultures at 0.1 MPa with TMAO; Lane 4, cultures at 30 MPa with TMAO. For the wild-type cell of QY27 and QY27 carrying control plasmid or *torZ*-silencing plasmid, the amount of proteins loaded was 6 μg for lanes 1 and 2, and 0.06 μg for lanes 3 and 4, respectively. For QY27 carrying *torA*-silencing plasmid, 6 μg protein was loaded for lanes 1 and 2, and 0.12 μg proteins for lanes 3 and 4. **(C)** The specific activity of TMAO reductase of different gene silencing strains cultivated at 0.1 and 30MPa. The Student’s *t*-test was performed, ^∗^*P* < 0.05, ^∗∗^*P* < 0.01, ^∗∗∗^*P* < 0.001, and ns represents no significant difference.

Compared to the cells cultivated at 0.1 MPa, the total TMAO reductase activity increased over twofold at 30 MPa. The addition of TMAO led to an increase over 30-fold at atmospheric pressure, whereas the simultaneous supplement of TMAO and the application of HHP enhanced the activity by approximately 76-fold (**Figure 4A**). By resolving the proteins on the native polyacrylamide gels, we further analyzed the effect of TMAO and HHP on different isozymes. As shown in **Figure 4B**, two bands displaying the activity of TMAO reductase were observed in the periplasmic fractions of QY27 cells cultivated at 0.1 MPa without TMAO (**Figure 4B**, QY27, lane 1). The activity of the upper-band was clearly enhanced, whereas the lower-band remained unchanged by applying HHP (30 MPa without TMAO) (**Figure 4B**, QY27, lane 2). When TMAO was present, the activity of the upper-band was significantly increased both under 0.1 and 30 MPa (**Figure 4B**, QY27, lanes 3 and 4). It should be noted that there was a remarkable increase in the TMAO reductase activity when TMAO is present in the culture medium (up to 76-fold). To guarantee the visibility of the activity bands from all of the samples, only a 1% amount of periplasmic proteins from the cells cultured with TMAO (**Figure 4B**, QY27, lanes 3 and 4) was loaded on the gel compared to those of the cultures without TMAO (**Figure 4B**, QY27, lanes 1 and 2). Thus, the lower band, whose intensity is not significantly induced by TMAO, cannot be visualized under this condition.

All together, these results demonstrate that the application of HHP alone increases the TMAO reductase activity of the upper-bands. We attempted to identify the TMAO reductase in the upper-band by mass spectrometry and found specific peptides of both TorA and TorZ. We could not exclude the contamination of the two bands when they were cut from the gels because the amount of the TMAO reductase is extremely low and their positions on the gels can only be visualized by oxygen-sensitive activity staining. Thus, a genetic analysis was performed as an alternative to determine which isoenzyme activity is enhanced by the HHP.

### Only TorA Was Induced by Elevated Pressure

To further dissect the effect of HHP on the two isozymes, we constructed *torA* and *torZ* gene silencing strains using the CRISPR-dCas9 system. The plasmid pCRISPRi carrying irrelevant DNA fragment targeting the *luxA* gene absent from QY27 genome was used as a control. It showed the same profile of TMAO reductase bands as the wild type strain at all the conditions tested (**Figure 4B**, QY27-i*luxA*). Silencing of the *torA* resulted in the disappearance of the upper-band at 0.1 MPa without TMAO (**Figure 4B**, QY27-i*torA*, lane 1). The addition of TMAO and HHP slightly augmented the activity of the upper-band but it was not comparable to the wild-type or QY27-i*luxA* (**Figure 4B**, QY27-i*torA*, lanes 2, 3, and 4). Silencing of the *torZ* had no visible effect on the upper band, but the lower-band was undetectable under all of the conditions (**Figure 4B**, QY27-i*torZ*). Therefore, the upper-band was mainly composed of TorA, whereas the lower-band was composed of TorZ.

The activity staining of the TMAO reductase on gels is a qualitative not quantitative method. We further compared the activity of TMAO reductase under 0.1 and 30 MPa in the three constructs by an enzyme assay. As in the wild-type strain, the activity increased approximately twofold at 30 MPa in QY27-i*luxA* and QY27-i*torZ*. However, it decreased at 30 MPa in QY27-i*torA* (**Figure 4C**). Thus, TorA was the dominant TMAO reductase in QY27 and was responsible for the HHP-induced TMAO reductase activity. This conclusion was also confirmed by quantitative RT-PCR and the transcription level of *torA* was significantly up-regulated under HHP, whereas *torZ* was slightly repressed (Supplementary Figure S9). In total, the expression of *torA* was under the control of both the substrate and HHP, whereas the regulation of *torZ* remained obscure because of its weak activity under all conditions.

### TorA Was Responsible for the TMAO-Improved Pressure Tolerance

**FIGURE 5:**
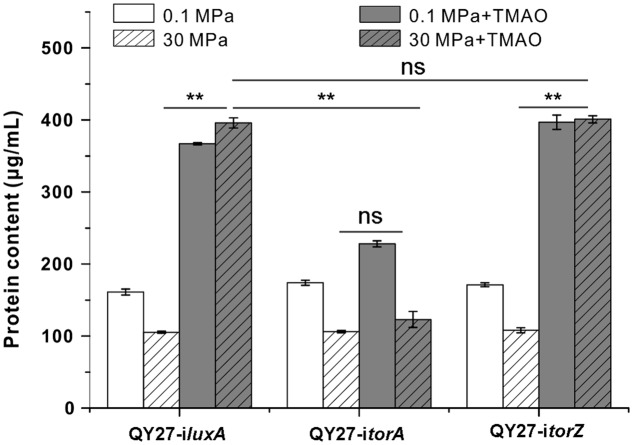
Contribution of TMAO reductase isozymes on the growth of QY27 at different conditions. The growth of QY27 carrying control plasmid (QY27-i*luxA*), *torA*-silencing strain (QY27-i*torA*) and *torZ*-silencing strain (QY27-i*torZ*) at different conditions. The blank and gray bars represent cultures at 0.1 and 30 MPa without TMAO, the white and gray bars with slash represent cultures at 0.1 and 30 MPa with TMAO, respectively. The biomasses were measured in the stationary phase. The Student’s *t*-test was performed, ^∗^*P* < 0.05, ^∗∗^*P* < 0.01, ^∗∗∗^*P* < 0.001, and ns represents no significant difference.

Gene silencing analysis revealed that HHP mainly increases expression and enzymatic activity of TorA and we further analyzed the contribution of the two isozymes to the growth of QY27 under different conditions. We found that both QY27-i*luxA* and QY27-i*torZ* showed similar growth profiles as the wild type QY27 strain. The elevated pressure hampered the growth when the cells were cultivated without TMAO, whereas supplementation of TMAO improved the growth, especially at 30 MPa (**Figure 5**). In contrast, the growth of QY27-i*torA* was no longer improved by the addition of TMAO and the TMAO-improved pressure tolerance was abolished as well. Consistent with the enzymatic assay, the growth experiment further confirmed that the isozyme TorA contributes mainly to the TMAO reduction in QY27 and is responsible for the phenotype of TMAO-improved pressure tolerance.

## Discussion

Microbial adaptation to HHP is a multifactor event (Abe et al., 1999; Oger and Jebbar, 2010; Tamegai et al., 2012; Jian et al., 2016). Transcriptomic and proteomic analyses revealed that alteration of the respiratory component according to hydrostatic pressure is a common strategy of the HHP adaption in deep-sea bacteria. The respiratory chain of piezophile *Shewanella violacea* DSS12 consists of the bc1-complex and cytochrome c oxidase at 0.1 MPa, whereas cytochrome c-551 and quinol oxidase exhibited higher pressure tolerance is utilized at 60 MPa (Ohke et al., 2013). Two sets of DMSO respiratory systems contribute differently to the growth of *S. piezotolerans* WP3 under different conditions (Xiong et al., 2016). Increasing evidence supports the bacterial anticipation of nutrient availability in the deep-sea to switch on the cognate energy metabolism pathway.

In this study, for the first time, we reported that TMAO facilitates the growth of QY27 under the HHP condition and confers on the piezo-sensitive strain a piezophilic-like phenotype. It has been reported that as one of the most important osmolytes in deep-sea fishes, TMAO protects the cells against low temperature and HHP (Yancey et al., 1982; Saad-Nehme et al., 2001; Zou et al., 2002; He et al., 2009; Petrov et al., 2012). Here, as revealed by real-time Raman spectra analysis, genetic and biochemical analysis, TMAO is utilized by QY27 as an electron acceptor of TMAO anaerobic respiration and TMAO reductase TorA is critical for the TMAO-improved pressure tolerance. Altogether, it can be concluded that unlike deep-sea fishes, the TMAO-improved pressure tolerance in deep-sea bacteria is mediated by TMAO respiration, and an HHP induced TMAO reductase is indispensable for this process.

We do not yet understand the physiological process involved in TMAO-improved pressure tolerance. One possible explanation is that TMAO promotes the growth of the HHP condition by providing the cell an alternative source of energy metabolism. At atmospheric pressure, TMAO is utilized from the beginning of growth onward (**Figure 3B**), and only one phase of growth was observed (**Figure 1B**). This suggests that TMAO is the only substrate of energy metabolism at this condition. Interestingly, a diauxic growth pattern was observed (**Figure 1B**, I and II) and TMAO is not consumed until approximately 20 h after inoculation at 30 MPa (**Figure 3B**). We propose a hypothesis to explain the biphasic growth and postponed utilization of TMAO under HHP conditions. QY27 is a facultative anaerobic bacterium and grows better in flasks with shaking than in syringes without shaking (Supplementary Figure S10), which indicates its preference of aerobic respiration using oxygen as electron acceptors. To study the HHP effect on their growth, the cells were incubated in syringes with a 2:3 air/media volume ratio without shaking. Under 0.1 MPa conditions, air slowly diffuses into the liquid media, which corresponds to microaerobic conditions and dissolved oxygen is quickly consumed and the cells use TMAO as electron acceptors from the early stage of growth. The application of HHP considerably increased the dissolved oxygen that supported the growth at the first phase (**Figure 1B**, I). When dissolved oxygen decreased below a certain level, the cells shift to TMAO anaerobic respiration, which leads to a high rate TMAO consumption and the second phase of growth (**Figure 1B**, II). During this period, the concentration of TMAO decreases at a higher rate compared to the cultures at 0.1 MPa. One reason is that at the end of the first phase of growth the cellular populations are more important than at the beginning of incubation, and the higher the cell numbers are the faster the TMAO is consumed. Another possible reason is that as shown by the enzymatic assay (**Figure 4B**), HHP supplementary enhanced the TMAO-induced activity of TMAO reductase, which may resulted in higher efficiency of TMAO respiration.

According to the current regulation model, the expression of TMAO reductase is strictly dependent on TMAO. The binding of TMAO intrigues conformational changes of periplasmic protein TorT and promotes the formation and stability of the TorT-TorS complex and thus activates the histidine kinase TorS. The response regulator TorR binds at the TTCATA motif in the promoter regions of *tor* gene clusters and activates the gene expression only when phosphorylated by TorS (Simon et al., 1995; Ansaldi et al., 2000; Bordi et al., 2004). HHP enhanced *torA* gene expression and TMAO reductase activity have only been previously reported in *P. profundum* SS9 and *P. phosphoeum* ANT-2200 (Vezzi et al., 2005; Le Bihan et al., 2013; Zhang et al., 2016). Here, we extend the same phenotype to *Vibrio*, which is another genus of Vibrionaceae. However, how the HHP induces the transcription of *torA* is unclear and whether it is mediated by the TorRS system or through an unknown regulator remains an enigma.

In the case of general regulation mechanisms, HHP affecting gene transcription has been observed in both piezosensitive and piezophilic bacteria (Ishii et al., 2005; Vezzi et al., 2005; Amrani et al., 2014). In deep-sea bacteria, several regulators and two component systems are suggested to be responsible for the induction of gene expression under HHP conditions such as the NtrBC system discovered in *S. violacea* DSS12 and the ToxRS system in *P. profumdum* SS9 (Nakasone et al., 1998, 1999, 2002; Welch and Bartlett, 1998; Bidle and Bartlett, 2001). In the regulatory model of the NtrBC system proposed by Nakasone et al. (2002) binding of the protein NtrC to the enhancer in the promoter region is increased under the HHP condition and thus up-regulates the expression of the *glnA* operon. The ToxRS two-component system is the first pressure sensor system identified in deep-sea bacteria. It is suggested that the transmembrane regulator ToxR could respond to the membrane changes resulting from HHP and regulates the transcription of two dozen genes. However, TMAO reductase does not belong to the ToxR regulon (Campanaro et al., 2012). It should be noted that the homologue of both NtrBC and ToxRS systems exists in piezo-sensitive bacteria, which indicates that the known regulatory system may evolve the ability to sense HHP in deep-sea species. Therefore, we cannot exclude the possibility that the TorSR system is involved in the HHP-induced *torA* expression. The sensing of pressure might occur independently or collectively at different levels. It is known that HHP executes an important effect on membrane fluidity (Oger and Jebbar, 2010) that might stabilize the TorT-TorS complex and trigger the auto-phosphorylation of TorS. Additionally, HHP might change the conformation of TorR and increase its affinity of binding to the *torA* promotor. We will focus on the construction of these mutants to analyze if and how TorR is involved in HHP-mediated *torA* up-regulation.

The ecological significance of the HHP induced TMAO reductase system in deep-sea bacteria remains unclear. TMAO is commonly found in the shallow water of the marine environment and even higher than that of DMSO and DMS (Gibb and Hatton, 2004). However, due to the difficulties in sampling and TMAO detection, the knowledge of occurrence and concentration of TMAO in the deep-sea environment is lacking. The fact that *Shewanella* species from different depths (from shallow water to over 9,000 m depth) all possess the TMAO reductase system (Wang et al., 2008; Aono et al., 2010) suggests that it is indeed a potential substrate for respiration of deep-sea species in the ambient environment. TMAO is highly abundant in deep-sea fish tissues (Yancey et al., 2014), and once released, the temporal and local high concentration of TMAO would be a precious nutrient for deep-sea bacteria. The cells with HHP induced TMAO reductase systems are prepared for the eventual events and are more competitive for life in nutrient deficient deep-sea habitats.

## Author Contributions

Q-JY, W-JZ, I-MC, and L-FW designed the study and analyzed the data. Q-JY, X-QQ, S-DZ, TJ, YC, and C-LS performed the experiments. X-GL, YC, C-LS, and HZ provided the technical support. W-JZ and L-FW wrote the manuscript. All authors read and approved the final manuscript.

## Conflict of Interest Statement

The authors declare that the research was conducted in the absence of any commercial or financial relationships that could be construed as a potential conflict of interest.

## Footnotes

1 https://www.ezbiocloud.net/
