# Supplementary material for: High Hydrostatic Pressure Inducible Trimethylamine N-Oxide Reductase Improves the Pressure Tolerance of Piezosensitive Bacteria Vibrio fluvialis
Source: Front Microbiol. 2018 Jan 9;8:2646. doi: 10.3389/fmicb.2017.02646 (PMC5767261; doi:10.3389/fmicb.2017.02646)
Supplement: Supplementary file 1 [file Data_Sheet_1.docx]

Supplementary Material

High hydrostatic pressure inducible Trimethylamine *N*-oxide reductase improves the pressure tolerance of piezosensitive bacteria *Vibrio fluvialis*

**Qun-Jian Yin, Wei-Jia Zhang,* Xiao-Qing Qi, Sheng-Da Zhang, Ting Jiang, Xue-Gong Li, Ying Chen, Claire-Lise Santini, Hao Zhou, I-Ming Chou, and Long-Fei Wu**

***Correspondence:** Wei-Jia Zhang: wzhang@idsse.ac.cn.

## Supplementary Figures

**
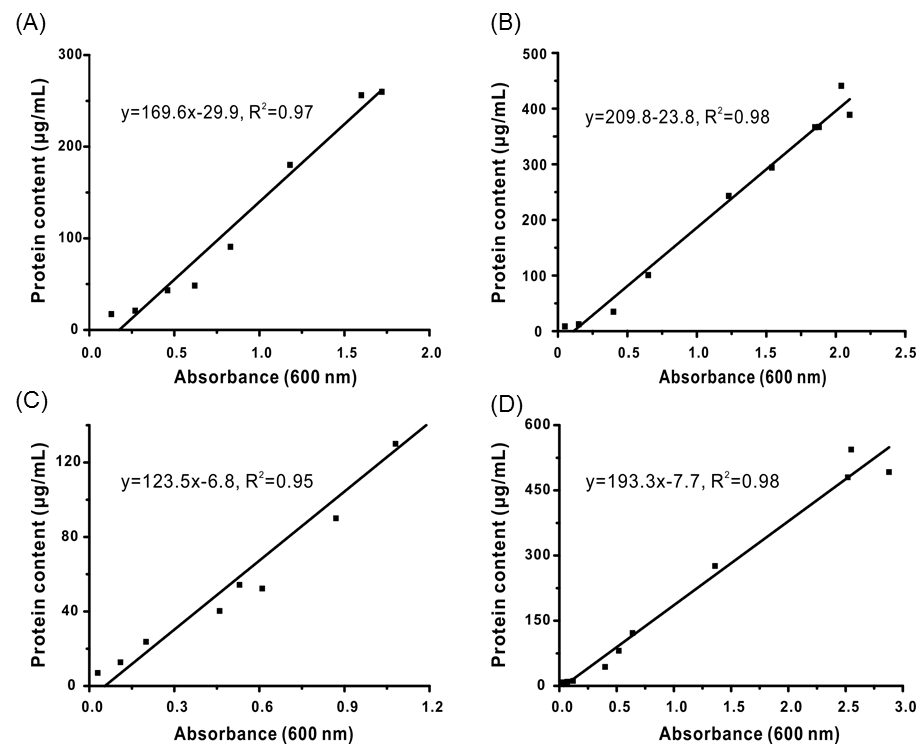
**

**Figure S1 |**The correlation between cell density and biomass of QY27 cultivated under different conditions.

**(A)**QY27 cultivated at 0.1 MPa without TMAO.**(B)**QY27 cultivated at 0.1 MPa with addition of TMAO.**(C)**QY27 cultivated at 30 MPa without TMAO.**(D)**QY27 cultivated at 30 MPa with addition of TMAO.The cell densities were analyzed by measuring the absorbance at 600 nm, the biomasses of same samples were then analyzed by quantifying the protein content.

**
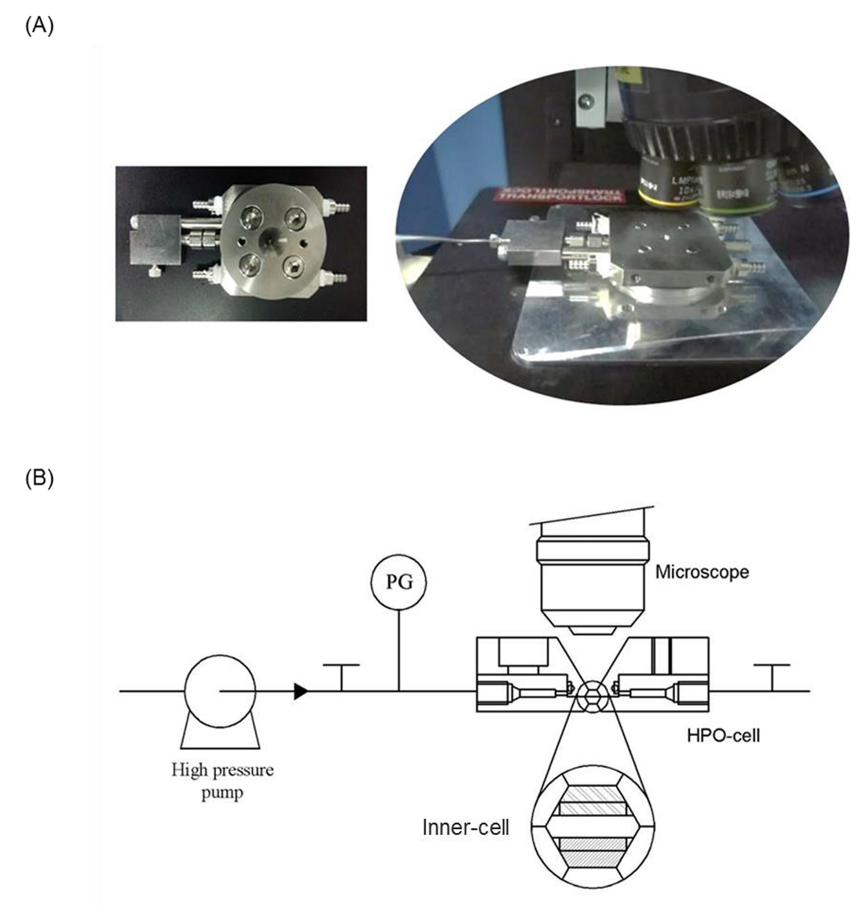
**

**Figure S2 |**The High-Pressure-Observation-cell (HPO-cell) for real time analysis of TMAO metabolism by Raman spectrometry.

**(A)** The photo of HPO-cell (left) and HPO-cell mounted on a Laser confocal micro Raman spectrometer (right).**(B)** The diagram of HPO-cell system and Raman spectrometer. PG: pressure gauge. QY27 was cultured in the inner-cell of the HPO-cell.

**
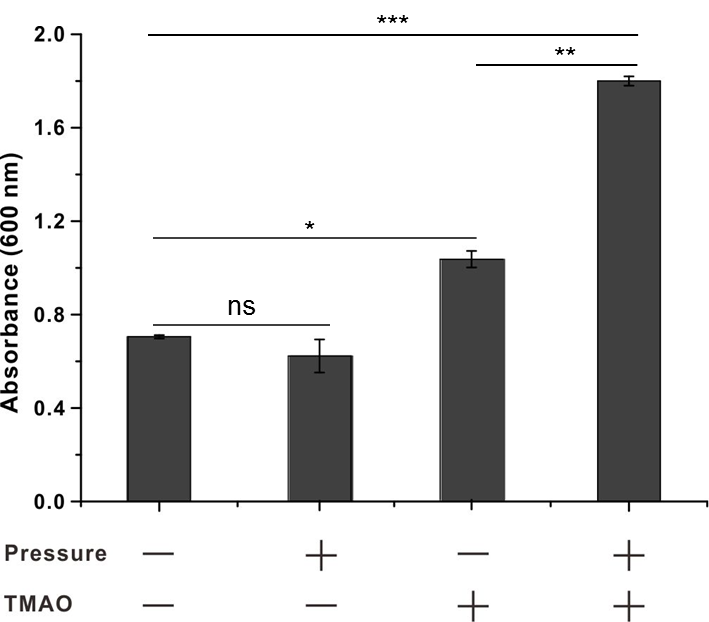
**

**FigureS****3 |**The growth of QY27at different conditions in minimal medium.

The cell density of QY27 cultivated in minimal medium at 0.1 MPa without TMAO (Lane 1, Pressure -, TMAO -), 30 MPa without TMAO (Lane 2, Pressure +, TMAO -),0.1 MPa with TMAO (Lane 3, Pressure -, TMAO +) and 30 MPa with TMAO (Lane 4, Pressure +, TMAO +). The cell densities were measured at stationary phase.The Student’s t-test was performed, ^*^P < 0.05, ^**^P < 0.01, ^***^P < 0.001, and ns represents nosignificantdifference.

**
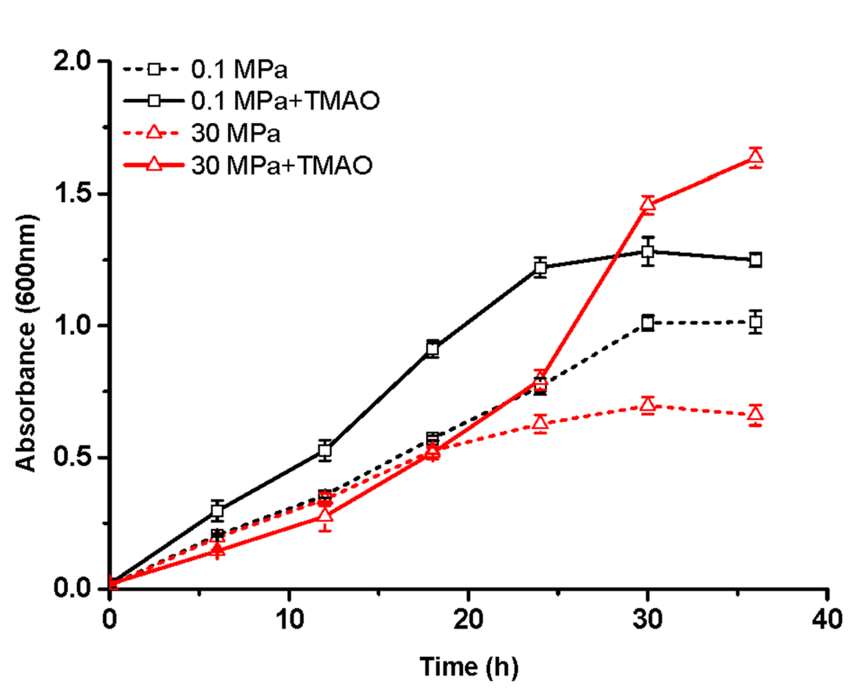
**

**FigureS4 |**The growth of*V. fluvialis* type strain ATCC33809 at different conditions.

The dash lines represent cultures without TMAO, the solid lines represent cultures with supplementation of TMAO. Lines in black represent cultures at 0.1 MPa, and lines in red represent cultures at 30MPa.

**
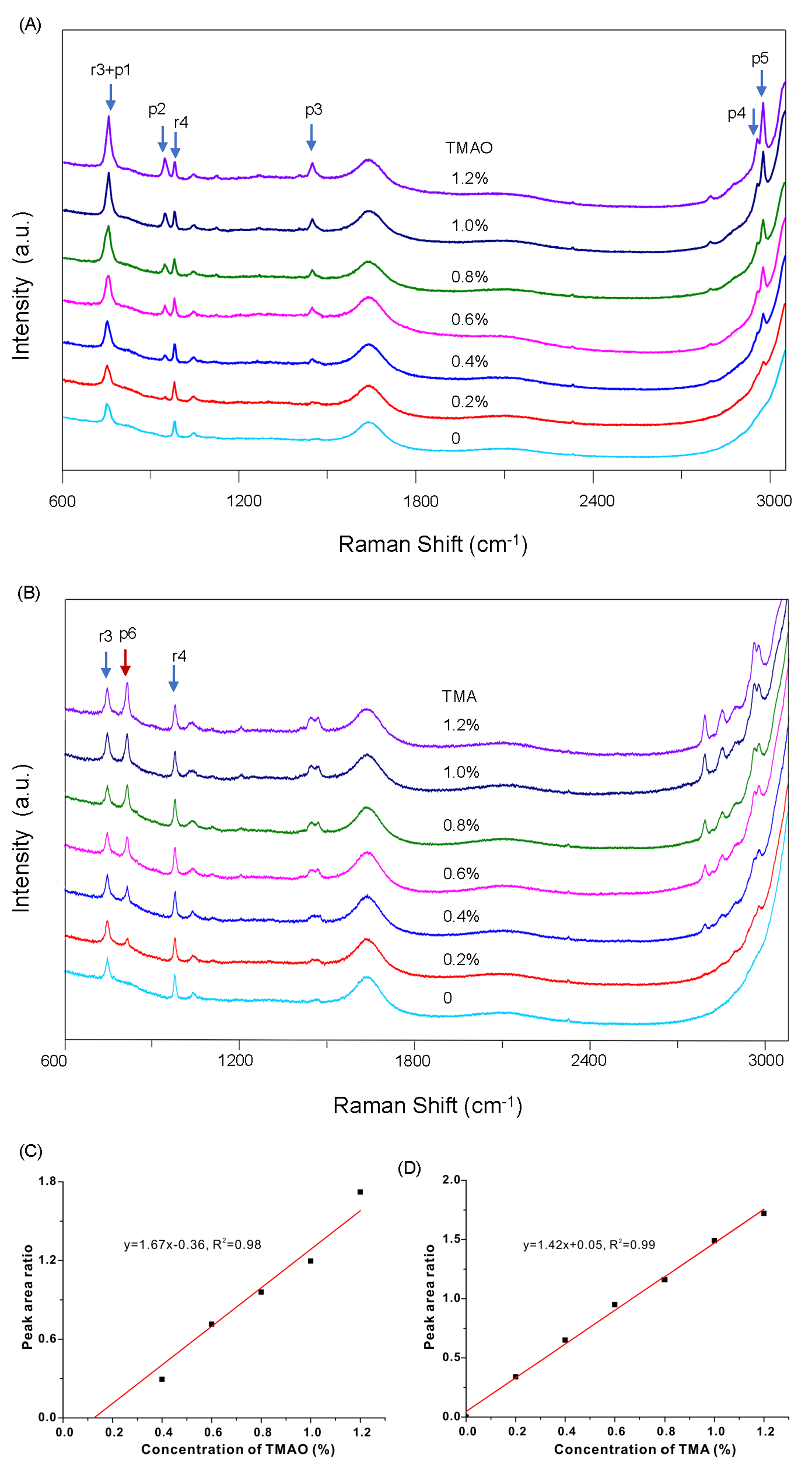
**

**FigureS5 |**The Raman spectra of minimal medium supplemented with different amount of TMAO and TMA.

The Raman spectra of TMAO **(A)** and TMA **(B)** in different concentrations. The concentrations of TMAO and TMAare indicated above each spectrum. The p2, p3, p4 and p5 are TMAO specific peaks, the p6 is TMA specific peak, and the r4 is unrelated peak derived from the medium. The peak r3+p1 consists unrelated peak r3 and TMAO specific peak p1.Linearity between concentration and the peak area ratio of TMAO- **(C)** or TMA-**(D)**specific peaks (p2 and p6, respectively) to reference peak r4.

**
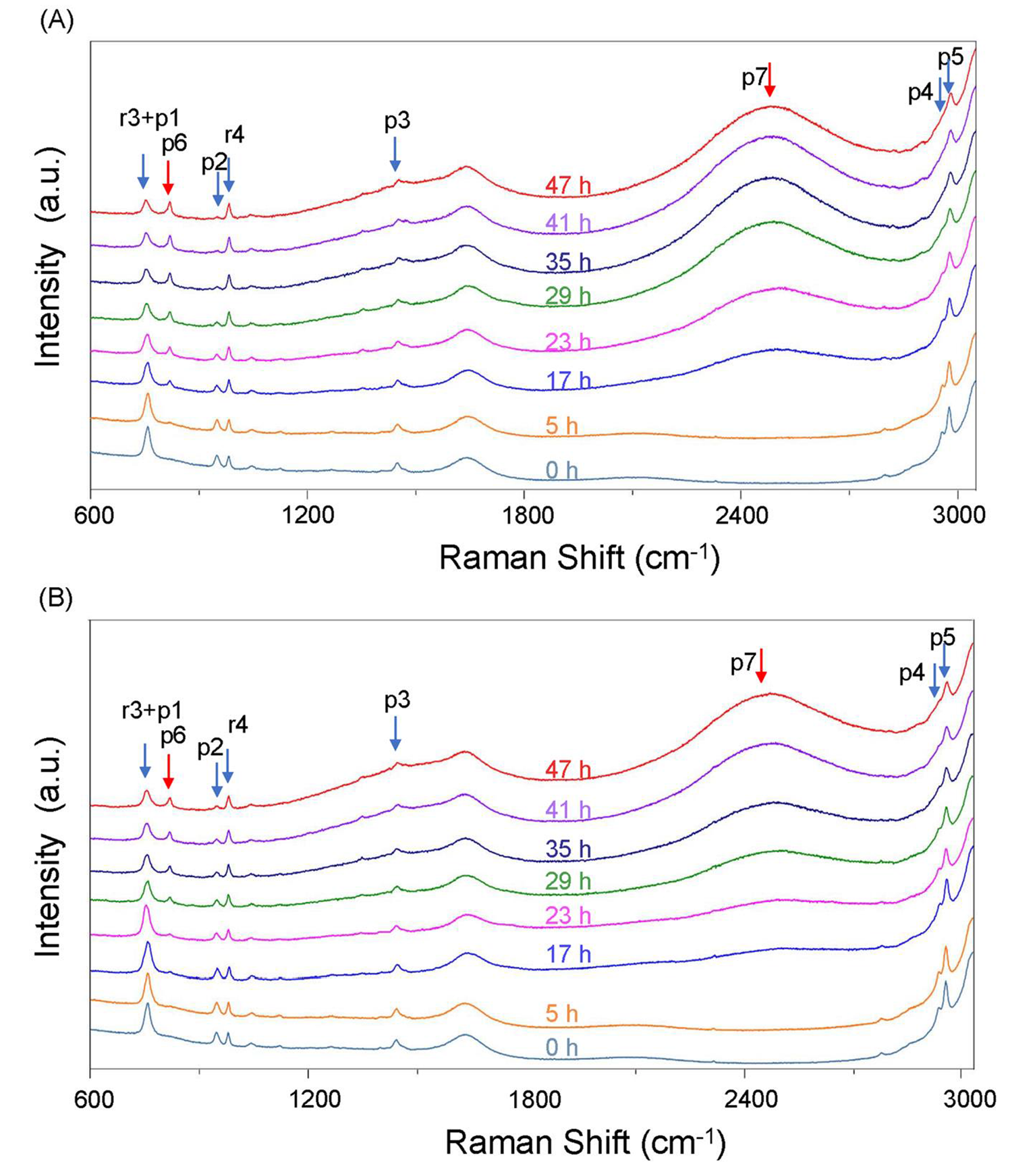
**

**FigureS6 |** Raman spectrometry analysis of TMAO metabolism at 0.1 MPa and 30 MPa.

The time course of Raman spectra of QY27 cultured in the minimal medium supplemented with 1% TMAO at 0.1 MPa **(A)**and 30 MPa **(B)**.Arrows mark the TMAO-specific peaks (p1 to p5), TMA-specific peak (p6) and unrelated peaks (r1 to r4).

**
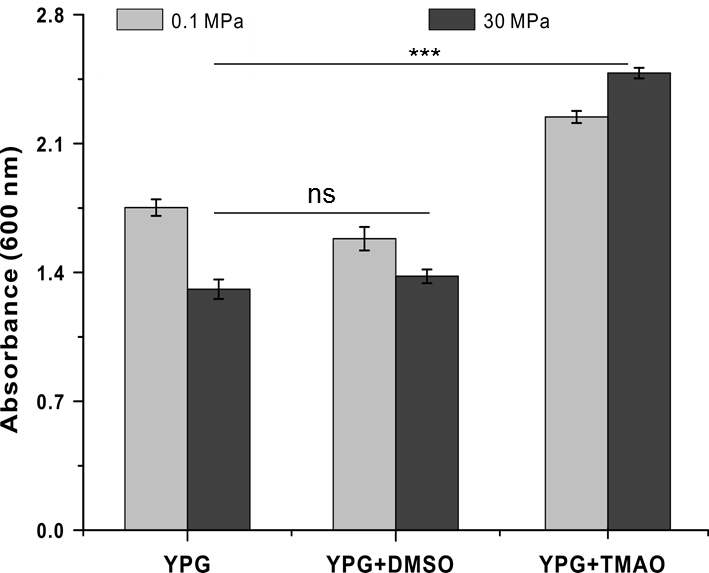
**

**Figure S7 |**The effect of TMAO and DMSO on the growth of QY27 at 0.1 MPa and 30 MPa.

The strain QY27 was cultivated at 0.1 MPa or 30MPa, in the YPG medium or YPG supplemented with TMAO or DMSO. The cell densities of different cultures were measured at stationary phase.The Student’s t-test was performed, ^*^P < 0.05, ^**^P < 0.01, ^***^P < 0.001, and ns represents nosignificantdifference.

**
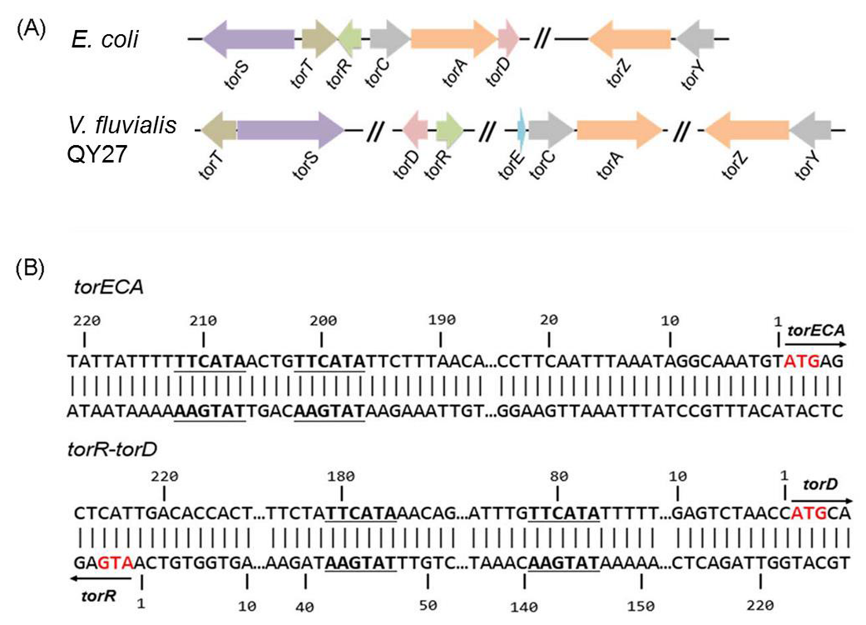
**

**Figure S8 |**TMAO reductase system of *Vibrio fluvialis*QY27.

The organization of TMAO reductase systems of *E. coli* and *V. fluvialis* QY27.

**
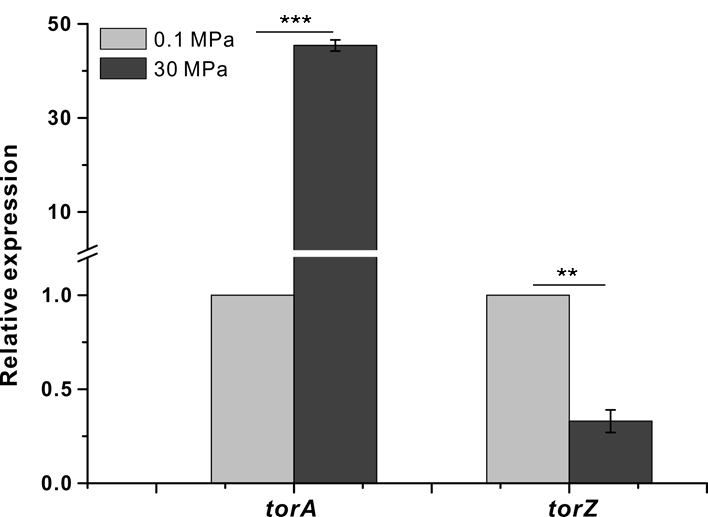
**

**Figure S9 |** The effect of pressure on transcription of TMAO reductase *torA* and *torZ*.

The transcription level of *torA* and *torZ* at 0.1 MPa were set as 1, respectively. The error bars indicate the standard deviation of three replicates.The Student’s t-test was performed, ^*^P < 0.05, ^**^P < 0.01, ^***^P < 0.001, and ns represents nosignificantdifference.


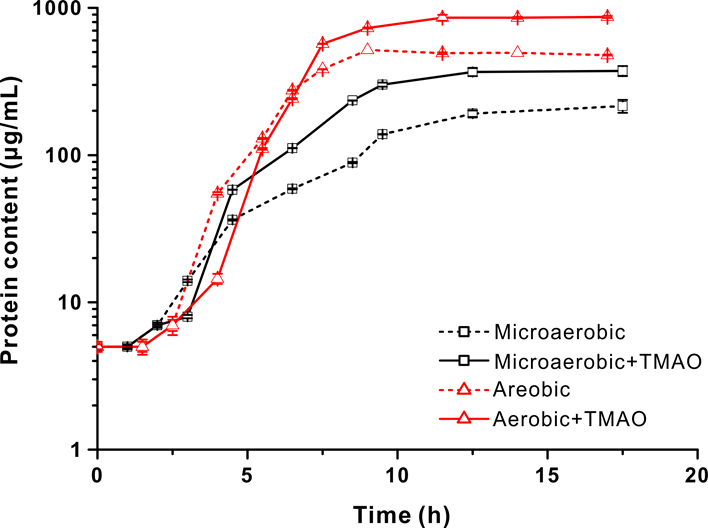


**Figure S10 |**The growth of*V. fluvialis* strainQY27at aerobic and microanarobic conditions.

The dash lines represent cultures without TMAO, the solid lines represent cultures with supplementation of TMAO. Lines in black represent cultures under microaerobic conditions, and lines in red represent cultures under aerobic conditions.
